# The Nicotine-Evoked Locomotor Response: A Behavioral Paradigm for Toxicity Screening in Zebrafish (*Danio rerio*) Embryos and Eleutheroembryos Exposed to Methylmercury

**DOI:** 10.1371/journal.pone.0154570

**Published:** 2016-04-28

**Authors:** Francisco X. Mora-Zamorano, Kurt R. Svoboda, Michael J. Carvan

**Affiliations:** 1 School of Freshwater Sciences, University of Wisconsin – Milwaukee, Milwaukee, Wisconsin, United States of America; 2 Joseph J. Zilber School of Public Health, University of Wisconsin – Milwaukee, Milwaukee, Wisconsin, United States of America; University Zürich, SWITZERLAND

## Abstract

This study is an adaptation of the nicotine-evoked locomotor response (NLR) assay, which was originally utilized for phenotype-based neurotoxicity screening in zebrafish embryos. Zebrafish embryos do not exhibit spontaneous swimming until roughly 4 days post-fertilization (dpf), however, a robust swimming response can be induced as early as 36 hours post-fertilization (hpf) by means of acute nicotine exposure (30–240μM). Here, the NLR was tested as a tool for early detection of locomotor phenotypes in 36, 48 and 72 hpf mutant zebrafish embryos of the non-touch-responsive *maco* strain; this assay successfully discriminated mutant embryos from their non-mutant siblings. Then, methylmercury (MeHg) was used as a proof-of-concept neurotoxicant to test the effectiveness of the NLR assay as a screening tool in toxicology. The locomotor effects of MeHg were evaluated in 6 dpf wild type eleutheroembryos exposed to waterborne MeHg (0, 0.01, 0.03 and 0.1μM). Afterwards, the NLR assay was tested in 48 hpf embryos subjected to the same MeHg exposure regimes. Embryos exposed to 0.01 and 0.03μM of MeHg exhibited significant increases in locomotion in both scenarios. These findings suggest that similar locomotor phenotypes observed in free swimming fish can be detected as early as 48 hpf, when locomotion is induced with nicotine.

## Introduction

In recent years, the zebrafish has emerged as a widely utilized aquatic model organism. The fecundity of this organism and the rapid development of its embryos enable scientists to perform large scale, phenotype-based screens in much shorter time frames than it would take to reproduce similar experiments in rodents [1]. With this premise in mind, a number of research groups have placed great interest in developing novel paradigms for behavioral phenotype-based screening in zebrafish embryos. Many behavioral paradigms have been devised for this model organism, among these there are paradigms to test locomotor output in response to visual [2,3], acoustic [4], tactile [5] and olfactory [6] cues. However, spontaneous locomotion remains arguably the most fundamental behavioral paradigm among the behavioral repertoire of zebrafish (*Danio rerio*) embryos and eleutheroembryos [7].

Historically, the use of behavioral screening in invertebrates and later in zebrafish was first utilized to detect variable genetic phenotypes that affected normal behavior [8]. However, toxicologists have adopted these methods for toxicity screening due to the broadness and robustness of the results that can be obtained [9]. Our study is not an exception to the aforementioned historical tendency to adapt screening assays from genetics to toxicology. The nicotine-evoked locomotor response (NLR) was first documented by Petzhold and collaborators [10] and although one of the observations of this group was that zebrafish did not respond to nicotine prior to 4 days of age, another research group had been able to use nicotine to induce spontaneous tail flicks in zebrafish embryos younger than 30 hours post-fertilization (hpf) [11]. These observations opened the question of whether the NLR could, in fact, be elicited in zebrafish embryos between 30 hours and 4 days of age.

Nicotine acts as an agonist of nicotinic acetylcholine receptors (nAChRs) and the NLR is triggered when acute doses of nicotine interact with nAChRs in the spinal cord [11,12], causing a sudden burst of locomotor activity in zebrafish embryos (S1 Fig and S1 File). Here we have taken advantage of the locomotion-inducing effects of nicotine to test the potential value of the NLR assay as a toxicological screening paradigm in 36 to 48 hpf zebrafish embryos—long before embryos develop sustained spontaneous swimming motor behaviors at 4–6 days post-fertilization (dpf).

Our need to develop an early embryonic locomotor screening paradigm arose from observations that zebrafish embryos exposed to methylmercury (MeHg) at concentrations significantly below lethal toxicity exhibited increased locomotor activity when analyzed at 6 dpf [13]. In the context of these observations, there was an interest to ascertain if increases in locomotor activity would be apparent at early developmental time points, if it was somehow possible to induce a locomotor output in fish much younger than 6 dpf.

Apart from the obvious benefit of saving time, an advantage of carrying out behavioral experiments in zebrafish embryos as early as 36–48 hpf is that, since the central nervous system (CNS) is not yet fully formed, any observed effects on locomotion would likely be attributable to more “primitive” anatomical structures such as the spinal neurons associated with the central pattern generator for swimming, the muscles, and the developing hindbrain [14], facilitating the interpretation of behavior-derived data.

To quantify the locomotor output of embryos during the NLR we utilized a cost-efficient approach comprised of a webcam-based video acquisition system paired with a free and open-source machine vision algorithm (S2 Fig). Together, the spontaneous swimming assay and the NLR assay, coupled with low-cost equipment and free and open-source machine vision software comprise a promising approach to carry out a simple diagnostic toxicity screening, which can later be supplemented with additional assays addressing more complex behaviors, if desired.

## Materials and Methods

### Fish husbandry

Wild type zebrafish breeding stocks were obtained from EkkWill Waterlife Resources (EK strain; Ruskin, Florida). The “macho” (*maco*) mutant zebrafish strain was acquired from the laboratory of Dr. Angeles Ribera, Anschutz Medical Campus at the University of Colorado. Both strains were maintained at 28°C on a 14h:10h light:dark cycle at the Children’s Environmental Health Sciences Core Center, located in the School of Freshwater Sciences of the University of Wisconsin—Milwaukee. The EK strain has been maintained in the laboratory for more than 15 generations. All of the animal protocols were approved by the Institutional Animal Care and Use Committee (IACUC) of the University of Wisconsin—Milwaukee.

EK embryos were obtained by breeding adult zebrafish in a ratio of two males to one female (10 males and 5 females in each breeding tank). Zebrafish of the *maco* strain were bred in a ratio of one male to one female (1 male and 1 female in each breeding tank). The adult fish would begin spawning at the onset of artificial dawn (9:00am), when the laboratory lights were automatically turned on. Embryos were raised for up to 6 dpf in Petri dishes (100mm × 15mm) containing E2 embryo medium (15mM NaCl, 0.5mM KCl, 1mM MgSO_4_, 150μM KH_2_PO_4_, 50μM Na_2_HPO_4_, 1mM CaCl_2_, 0.7mM NaHCO_3_; pH 7.2) at a density of 200 embryos per dish; the embryo medium was exchanged daily.

### Dose-dependent effects of nicotine on the NLR

Four nominal doses of nicotine (30, 60, 120 or 240μM; Sigma, St. Louis, MO) were used to assess the NLR in zebrafish embryos in two different stages of early development (36 and 48 hpf). The embryos were manually dechorionated, and then transferred into a recording vessel (89mm × 89mm × 25mm, white semitransparent rubberized polystyrene weighing boat; Cole-Parmer, Vernon Hills, IL) containing 10ml of a nicotine solution. The embryos (n = 12 per vessel) were transferred with a fine tip glass pipette, ensuring that the clean medium necessary to carry the embryos over was kept consistent and to a minimum (~1ml) to avoid altering the concentration curve of the nicotine solutions. The embryos were video recorded as soon as the tip of the glass pipette touched the nicotine medium. None of the NLR assays discussed henceforth included an acclimation period prior to video recordings due to the rapid onset of this response (*i*.*e*. the NLR occurs within seconds). However, we do not believe that the lack of acclimation affects the NLR.

### Video recording apparatus and behavior quantification

The video recording apparatus consisted of a manifold holding four Logitech C920 web cameras pointing downwards into a Plexiglas tray that holds four weigh boats. Underneath the apparatus, a flat 22” Acer P221W computer monitor was used as a light source, which provided 58 lux of constant illumination; a sheet of velum paper was used as a diffusing filter. In order to block extraneous light and visual stimuli, the whole apparatus was surrounded by a custom made black polyethylene enclosure (S2 Fig). All video recordings were streamed to a remote computer (Lenovo T410; Intel Core i5 CPU @ 2.53GHz, 4.00 GB RAM) at a resolution of 960×720 pixels and at a frame rate of 30 frames per second using the MATLAB (The MathWorks, Inc.; Natick, MA) image acquisition toolbox. The NLR of the embryos was tracked using a free and open-source machine vision algorithm (ctrax [15]) and tracking errors were manually corrected using the “fixerrors” MATLAB toolbox provided by the ctrax developers. The raw trajectory data was imported to a custom Microsoft Excel macro (Microsoft; Redmond, WA) to calculate the maximum speed (mm s^-1^) and the “latency of response” (time required to reach maximum speed; s) of each individual embryo. Twelve embryos were analyzed for each condition tested.

### Modulation of the NLR by chronic low-dose exposure to nicotine

It has been observed that the tail beat frequency of zebrafish embryos during the NLR can be modulated if the embryos are reared in a low dose of nicotine (1μM) for 12 hours prior to the NLR assay (Dr. Matthew E. Wolter, Svoboda laboratory, University of Wisconsin—Milwaukee; personal communication). Here we investigated if this observation translated into differences in maximum speed and latency of response. Zebrafish embryos were raised in clean embryo medium for 36 hours and then transferred into media containing 0, 0.5 or 1μM of nicotine for an additional 12 hours. The now 48 hpf embryos were submitted to the NLR assay using 120μM of nicotine to trigger the response. Twelve embryos were analyzed for each of the 12 hour low dose nicotine pre-treatment regimes (12 embryos were evaluated per treatment).

### Analysis of the NLR in *maco* zebrafish mutants

Zebrafish of the *maco* strain do not exhibit a touch response due to impaired sodium channel action potentials [16]. The known locomotor impairment in this mutant strain was utilized as a premise to investigate the potential of the NLR assay to discriminate between the maximum speed of embryos with and without overt locomotor abnormalities. Embryos of the *maco* strain were raised to three different developmental stages (36, 48 and 72 hpf) in clean embryo medium and then manually dechorionated. Before carrying out the NLR assay, a quick phenotypic screening on the embryos was done by performing a touch response test under a stereoscopic microscope (Olympus SZ61; Olympus Life Science Solutions; Center Valley, PA). After separating mutant embryos from their siblings without a phenotype, the two groups of embryos were tested with the NLR assay using a 120μM nicotine solution to trigger the response. Three replicates of this experiment were performed for each of the aforementioned conditions (approximately 10 embryos per replicate).

### MeHg exposure

To assess the effects of MeHg exposure on both the free swimming and NLR of zebrafish, ≤2 hpf embryos were treated with MeHg chloride (MeHg; Sigma-Aldrich Co.; St. Louis, MO) using ethanol (0.01%) as vehicle at nominal concentrations of 0, 0.01, 0.03 and 0.1μM in E2 embryo medium; a vehicle control solution (0μM MeHg) was prepared diluting 0.01% ethanol in E2 medium. The morning after the exposures had begun (24 hours of exposure), the embryos were rinsed three times with clean E2 embryo medium (not supplemented with MeHg or ethanol) to eliminate residual MeHg in solution. This procedure was performed to ensure that no significant amounts of MeHg would continue to be absorbed through the chorions after 24 hours of exposure. The embryos were then raised in clean E2 medium until needed for locomotor assessment.

### Free swimming of MeHg exposed zebrafish

Newly spawned embryos were exposed to MeHg as described above, then raised to 6 dpf to assess the rate of travel (distance traveled in 5 minutes; mm 5min^-1^) and activity (% of time active) of the free swimming eleutheroembryos. Prior to video recordings, the fish were allowed to acclimate for 5 minutes. A total of 120 fish per dose (10 fish per recording vessel; 12 vessels per dose) were video recorded and their locomotion was analyzed.

### NLR of MeHg exposed 48 hpf zebrafish

The NLR assay was performed in MeHg-exposed 48 hpf embryos. 10 embryos were analyzed per recording vessel; 120μM of nicotine was used to trigger the NLR. The maximum speed, latency of response and distance traveled (in 2 minutes) was calculated for 50 embryos for each dose tested.

### Statistical analyses

Statistical analyses were conducted with SigmaPlot software version 11.0 (Systat Software; San Jose, CA). All data was tested for normality using the Shapiro—Wilks test. If the data was found to be normally distributed, a one-way ANOVA was performed, subsequently a *post hoc* multiple pair-wise comparison between exposure groups was carried out with the Holm—Sidak method. Non-normal data was analyzed with ANOVA on ranks using the either the Mann-Whitney or the Kruskal—Wallis H test (H values are reported in all statistics that required the Kruskal—Wallis H test), *post hoc* multiple pair-wise comparisons between exposure groups were performed with Tukey’s method.

## Results

### Dose-dependent effects of nicotine on the NLR

**Fig 1:**
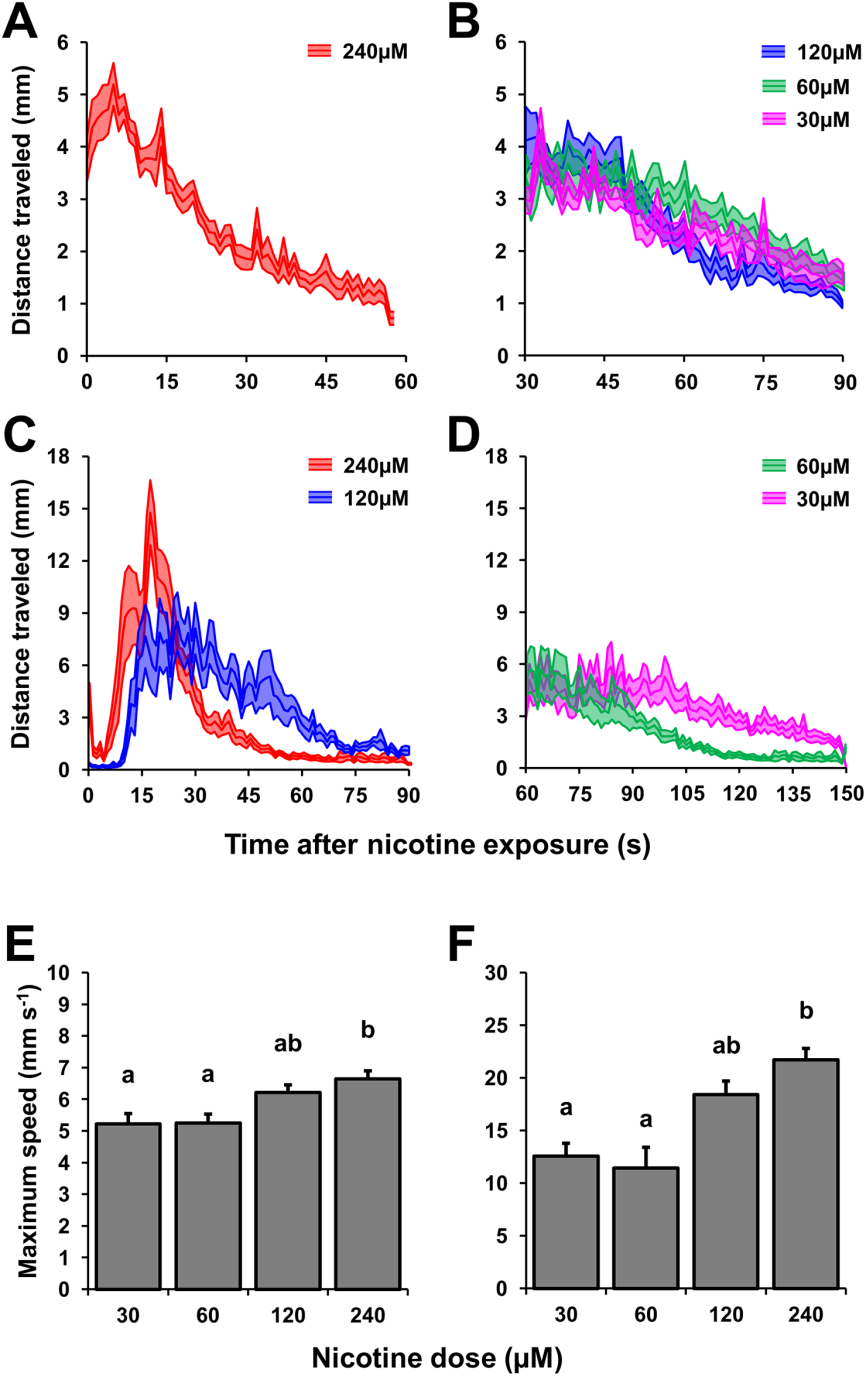
Nicotine-evoked locomotion dose response curves in 36 and 48 hpf zebrafish embryos. Nicotine dose response curves in 36 hpf (A and B) and 48 hpf (C and D) zebrafish embryos during the NLR. The NLR of 36 hpf embryos triggered by 240μM of nicotine was characterized by a significantly higher maximum speed than the observed in the rest of the doses tested (E). Similarly, 240μM of nicotine triggered a significantly higher maximum velocity in 48 hpf embryos (F). The effects of a control solution with no nicotine is not represented in the graphs due to the fact that 36 and 48 hpf embryos simply do not exhibit any swimming output in the absence of nicotine. The notation above the bar graphs represent pair-wise comparisons.

In this whole study, every one of the ~512 embryos tested with the NLR paradigm exhibited locomotor output in response to acute nicotine exposure (out of these ~512 fish, 90 were *maco* mutants and 150 were exposed to MeHg), which highlights the effectiveness and reliability of the NLR paradigm. Embryonic developmental stage had an effect on the NLR. 36 hpf embryos achieved overall lower maximum speeds than 48 hpf embryos in all nicotine concentrations tested (n = 12 embryos, P<0.001; 30μM, P<0.001; 60μM, P = 0.017; 120μM, P<0.001; 240μM, P<0.001). The NLR was affected by nicotine dose; 240μM of nicotine triggered a significantly higher maximum velocity in both 36 hpf (H = 13.4, n = 12 embryos, P = 0.004) and 48 hpf embryos (H = 38.1, n = 12 embryos, P<0.001), relative to embryos exposed to 30, 60 and 120μM of nicotine, both in 36 and 48 hpf embryos (Fig 1). High nicotine doses also reduced the latency of the embryos to reach their maximum velocity; 36 hpf embryos exposed to 240μM of nicotine reached their maximum velocities quicker than embryos exposed to 30, 60 and 120μM (H = 29.9, n = 12 embryos, P<0.001). Likewise, 240 and 120μM of nicotine decreased the latency to reach maximum velocity in 48 hpf embryos, compared to embryos exposed to 30 and 60μM (H = 38.1, n = 12 embryos, P<0.001; Table 1). From a practical standpoint, 36 hpf embryos were more difficult to track with the ctrax algorithm due to their lack of pigmentation and significantly slower NLR; both of these factors can complicate the differentiation between moving embryos and the background. Furthermore, higher doses of nicotine facilitated the analysis of the NLR, given that this reduces the time that the embryos remain immotile and aggregated during the first few seconds of the response, hence reducing mismatches and ambiguities. In 48 hpf embryos, a dose of 120μM of nicotine delivered a satisfactory NLR that was not significantly different to the NLR evoked by 240μM, for this reason we concluded that the optimal experimental conditions for the NLR assay as a screening tool would be to trigger the response with 120μM of nicotine utilizing 48 hpf embryos.

**Table 1:** NLR dose response in 36 and 48 hpf zebrafish embryos. Values are presented as the mean ± SE. Means sharing the same superscript are not significantly different from each other P < 0.05.

	36 hpf zebrafish embryos		48 hpf zebrafish embryos	
Nicotine dose (μM)	Maximum speed (mm s^-1^)	Latency of response(s)	Maximum speed (mm s^-1^)	Latency of response (s)
30	5.29±0.37^a^	43.08±13.62^a^	12.55±1.24^a^	83.50±4.76^a^
60	5.25±0.28^a^	48.92±11.72^a^	11.42±1.99^a^	69.58±2.54^a^
120	6.21±0.24^ab^	38.17±7.33^a^	18.43±1.27^ab^	29.50±4.13^b^
240	6.64±0.26^b^	6.92±4.50^b^	21.72±1.08^b^	17.92±1.95^b^
**ANOVA on ranks (Kruskal**–**Wallis test)**				
***H***	*13*.*4*	*29*.*9*	*22*.*5*	*38*.*1*
***P***	***0*.*004***	**<*0*.*001***	**<*0*.*001***	**<*0*.*001***

### Modulation of the NLR by chronic low-dose exposure to nicotine

Rearing 48 hpf zebrafish embryos in 1μM of nicotine for 12 hours prior to the NLR test resulted in significantly lower maximum velocities (H = 17.41, n = 12 embryos, P<0.001), coupled with a higher latency to reach maximum speed (H = 23.56, n = 12 embryos, P<0.001), relative to embryos reared in 0 and 0.5μM of nicotine. Embryos reared in 0.5μM of nicotine did not exhibit significant changes in maximum speed or distance traveled throughout 90 seconds of observation; however, they reached maximum velocities significantly quicker than the embryos from the 0 and 1μM nicotine exposure groups (H = 23.56, n = 12 embryos, P<0.001) (Table 2).

**Table 2:** Modulation of the NLR in 48 hpf zebrafish embryos by chronic, low-dose exposure to nicotine during development. Values are presented as the mean ± SE. Means sharing the same superscript are not significantly different from each other P < 0.05.

Embryonic nicotine exposure dose (μM)	Maximum speed (mm s^-1^)	Latency of response (s)	Distance traveled (mm 90s^-1^)
0	21.72±1.08^a^	17.92±1.95^a^	299.63±16.08^a^
0.5	25.69±1.53^a^	6.17±0.89^b^	257.31±18.30^a^
1.0	16.58±1.10^b^	37.25±6.18^a^	310.66±26.19^a^
**ANOVA on ranks (Kruskal**–**Wallis test)**			
***H***	*17*.*41*	*23*.*56*	*3*.*36*
***P***	**<*0*.*001***	**<*0*.*001***	***0*.*187***

### Analysis of the NLR in *maco* zebrafish mutants

**Fig 2:**
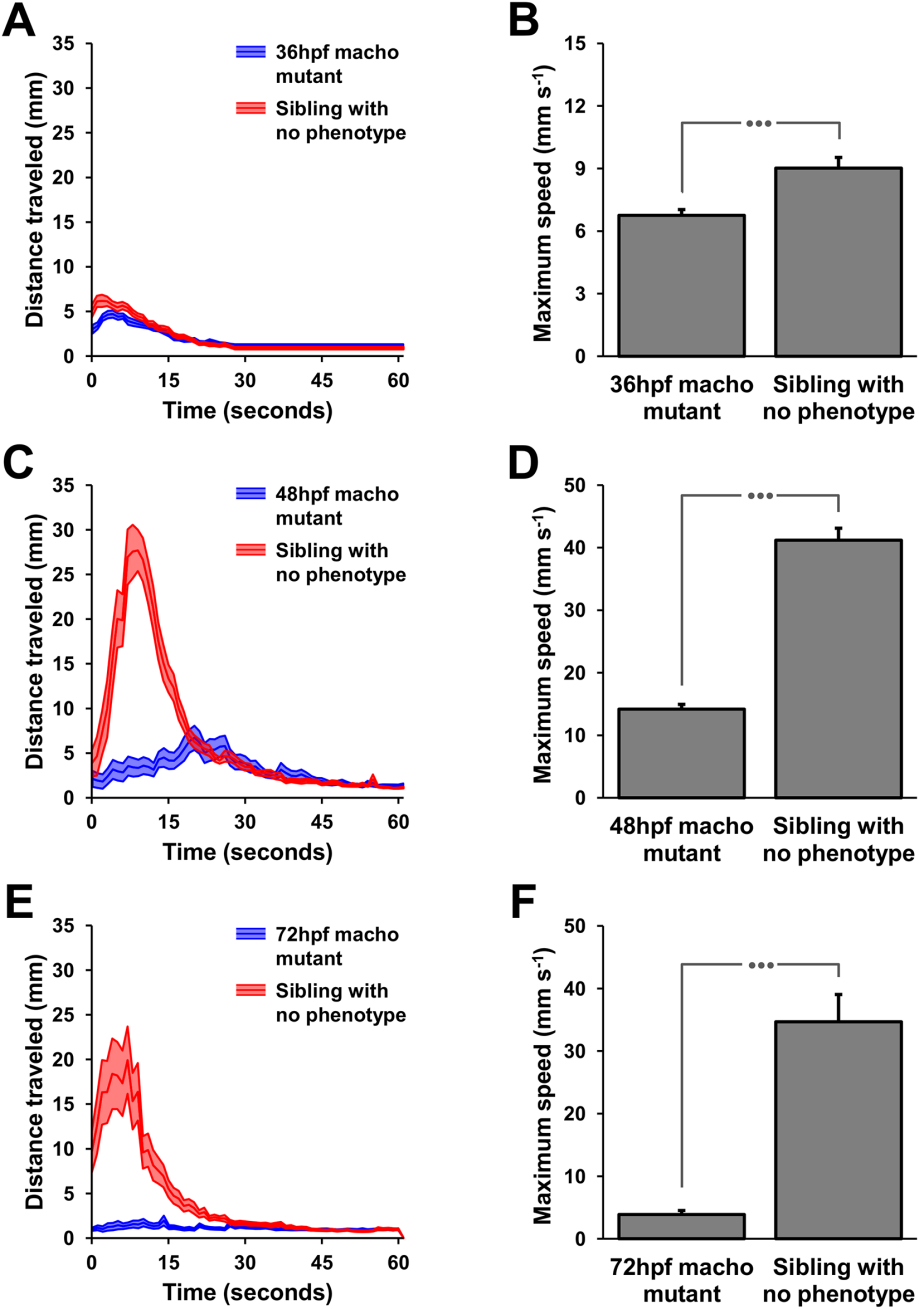
Validation of the NLR assay: analysis of the non-touch-responsive *maco* mutant zebrafish strain. The NLR assay was successful at discriminating mutant embryos from their non-mutant siblings. The locomotor activity of 36 hpf embryos triggered by 120μM of nicotine was significantly different between mutants and non-mutant embryos (A) as demonstrated by the comparison of the average maximum speed of mutant and non-mutant embryos (B). The difference between mutants and non-mutants became progressively more apparent in 48 hpf (C and D) and 72 hpf (E and F) embryos. The three dots above the graphs in panels B, D and F represent P <0.001.

Zebrafish embryos of the *maco* mutant strain were utilized due to their known locomotor phenotype to test the effectiveness of the NLR assay to identify overt locomotor abnormalities. The NLR assay was successful at discriminating mutant embryos from their non-mutant siblings. All zebrafish mutants of the *maco* strain tested with the NLR paradigm had significantly lower maximum speeds (Mann—Whitney U statistic; 36 hpf, T = 390, n_small_ = 23, n_large_ = 27, P<0.001; 48 hpf, T = 300, n_small_ = 24, n_large_ = 24, P <0.001; 72 hpf, T = 552, n_small_ = 30, n_large_ = 30, P <0.001; Fig 2).

### Free swimming and NLR of MeHg exposed eleutheroembryos and embryos

**Fig 3:**
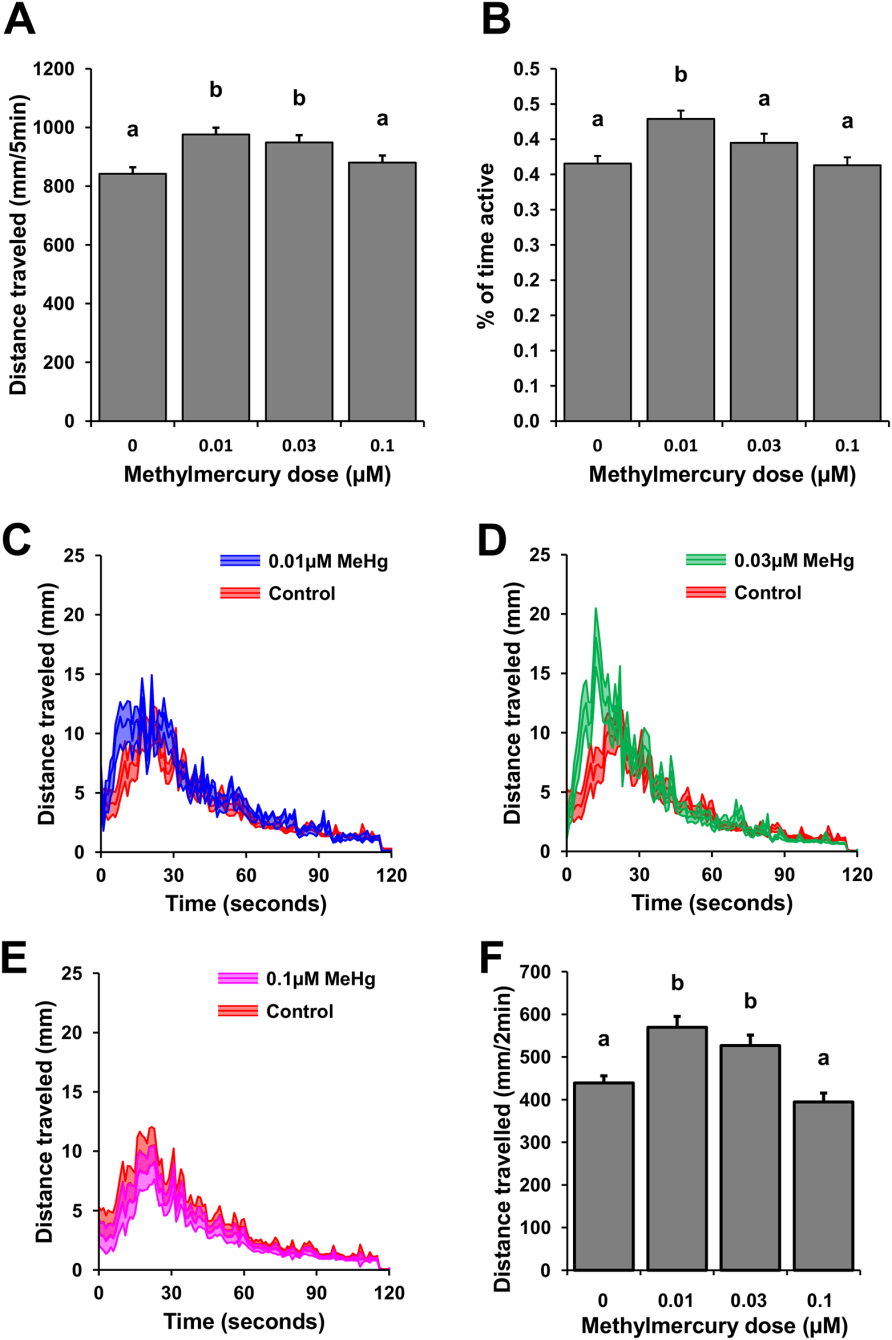
Spontaneous swimming of 6 dpf MeHg-exposed zebrafish eleutheroembryos and the NLR of MeHg-exposed 48 hpf embryos. The spontaneous-swimming assay elucidated subtle yet significant (P<0.001) increases in the total distance travelled (mm in 5 minutes) of free swimming 6 dpf zebrafish exposed to 0.01 and 0.03μM of MeHg (A). Eleutheroembryos exposed to 0.01μM MeHg as embryos also had an increased activity (% of time active) relative to all other doses (B). The NLR assay was conducted on 48 hpf embryos exposed to 0, 0.01, 0.03 and 0.1μM; the activity curves of all MeHg-exposed embryos were compared to the control (C through E). As observed in 6 dpf eleutheroembryos, 48 hpf zebrafish embryos exposed to 0.01 and 0.03μM of MeHg exhibited an increase in distance traveled during the analysis period (F).

Zebrafish eleutheroembryos (6 dpf) exposed to 0.01 and 0.03μM of MeHg exhibited a significantly increased rate of travel during the five minutes of activity tracking (H = 26.49, n = 120 embryos, P<0.001, Fig 3A). Additionally, eleutheroembryos exposed to 0.01μM of MeHg were more active than the embryos of the rest of the exposure groups (H = 26.71, n = 120 embryos, P<0.001, Fig 3B). Similar to the results observed in 6 dpf eleutheroembryos, 48 hpf zebrafish embryos exposed to 0.01 and 0.03μM of MeHg had an increased rate of travel (F_3,196_ = 12.82, n = 50 embryos, P<0.001) and maximum speeds (F_3,196_ = 11.9, n = 50 embryos, P<0.001) compared to the 0 and 0.1μM exposure groups (Fig 3C–3F; Table 3).

**Table 3:** Effect of MeHg on the NLR of 48 hpf zebrafish embryos. Values are presented as the mean ± SE. Means sharing the same superscript are not significantly different from each other P < 0.05.

Embryonic MeHg exposure dose (μM)	Maximum speed (mm s^-1^)	Latency of response (s)	Distance traveled (mm 2min^-1^)
0	33.06±1.47^a^	19.26±1.70^a^	439.01±16.77^a^
0.01	41.06±1.48^b^	18.92±1.65^a^	569.29±25.84^b^
0.03	43.27±1.90^b^	17.94±1.74^a^	526.67±24.57^b^
0.10	31.64±1.80^a^	14.50±1.45^a^	394.46±20.90^a^
**ANOVA**			
***F***_***3*,*196***_	*11*.*92*	*1*.*76*	*12*.*82*
***P***	**<*0*.*001***	***0*.*156***	**<*0*.*001***

## Discussion

Our approach is an adaptation of the methods published by Schneider and collaborators [17], where the NLR was utilized as a behavioral paradigm for drug development or screening in 5 and 6 dpf zebrafish. That work built upon the previous work of Petzhold and collaborators [10], who initially used nicotine-evoked locomotion to identify genes in the zebrafish that were potentially involved with nicotine addiction.

Here, we adopted this paradigm for use in toxicology; our protocol integrated a custom behavior observation apparatus, a free and open-source machine vision algorithm and most importantly, the assessment of the NLR in zebrafish embryos as young as 36 hpf. The possibility to assess the NLR in such early stages of development makes the NLR assay a promising tool to investigate the early ontogeny of locomotor development in zebrafish.

In order to validate the value of the NLR assay for early toxicological screening in zebrafish embryos, our experimental design addressed three main areas of interest. First we tested the effect of triggering the NLR with varying acute doses of nicotine, as well as the effect of chronic low dose nicotine exposure on the NLR. Secondly, we evaluated the NLR assay in a simple genetic phenotype-based screening, using the non-touch-responsive *maco* zebrafish mutants. Third and lastly, we evaluated the effect of MeHg—a well acknowledged neurotoxicant—on the NLR and free swimming of zebrafish embryos and eleutheroembryos.

The first set of experiments demonstrated important qualities of the pharmacology of nicotine exposure; analogous to the observations of Thomas and collaborators [11] where acute exposure to nicotine resulted in a dose-dependent increase in locomotor output of zebrafish embryos. The majority of those experiments carried were performed in embryos <30 hpf and the frequency of embryonic tail bends per minute were manually quantified. It is relevant to emphasize that at this age, zebrafish embryos did not exhibit any forward propulsion, only spontaneous tail bends. In the case of our study, the analysis of the NLR was performed in 36 to 72 hpf embryos, and at these developmental stages, the embryos exhibited a strong swimming burst in response to acute nicotine exposure, which were quantified by means of a machine vision algorithm.

Thomas and collaborators also observed an apparent “desensitization” of the NLR in embryos subjected to a chronic exposure to nicotine. Similar to these reports, our study identified a reduction in the locomotor output during the NLR of embryos exposed to 1μM of nicotine for 12 hours, however embryos treated with 0.5μM of nicotine for 12 hours exhibited higher maximum velocities and reached these maximum velocities quicker compared to embryos reared in clean embryo medium. It has been proposed that reductions in the NLR in embryos exposed chronically to nicotine (1–30μM) could be attributed to nicotine binding to nAChRs and acting as an antagonist of these receptors during this first exposure, or by nAChR desensitization. In either scenario, the behavioral effect of a subsequent acute nicotine exposure would be greatly diminished [11]. In contrast to this, nicotine exposure causes calcium and or sodium entry into the neuron causing it to become “excited” closer to threshold for firing action potentials. If the receptors do not desensitize, and if some nAChRs were still available to participate in the NLR, a second exposure to nicotine would then cause those neurons to fire action potentials quicker. This likely explains why NLRs of embryos pre-exposed to 0.5μM nicotine exhibited quicker onsets compared to NLRs in control embryos.

As originally postulated by Petzhold and collaborators [10], the NLR assay proved to be an excellent genetic screening tool. This was illustrated by our success at differentiating between mutant and non-mutant *maco* zebrafish siblings. One important reason for utilizing the *maco* strain in our experiments is that fish from this strain are capable of moving in contrast to fish from a fully paralytic strain, such as “sofa potato”. However, *maco* zebrafish do not exhibit a touch response after 27 hpf. This lack of a touch response is attributed to abnormalities in the failure of Rohon—Beard (RB) cells to fire action potentials [16]. Our results showed decreased locomotor responses in *maco* fish assayed in the NLR even at 72 hours of age, a point of development where dorsal root ganglion (DRG) neurons are thought to functionally replace the transient RB neuron [18].

In order to understand why the older *maco* mutants do not exhibit a robust response to nicotine, an appreciation of how nicotine activates locomotion is needed. When developing embryos are exposed to nicotine, they exhibit alternating bends of the musculature reminiscent of swimming [11]; this is an “embryonic swim-like behavior”. When the concentration of nicotine is low, the embryos barely move. When the concentration is increased, the onset of activity is quicker, and the bend rate is faster. In these young embryos, the nicotine has to penetrate the skin and musculature to get to the neurons within the central nervous system to activate the motor output. If embryos are manipulated in a way to reduce expression of specific nAChRs in spinal cord, nicotine exposure does not activate a motor output [12]. If the hindbrain is separated from the spinal cord, (*i*.*e*. if the head is cut and separated from the tail) the severed tails still swim when exposed to nicotine. Collectively, these data indicate that spinal nAChRs activated by nicotine can initiate a motor output that resembles swimming. Consistent with these observations, it is known that RB neurons express nAChRs [12,19,20]. Importantly, nAChRs in zebrafish are also expressed by DRG neurons (Svoboda laboratory, unpublished observations) and spinal interneurons [21].

In 36 hpf *maco* mutants, nicotine was able to evoke a fairly robust motor output, but this was reduced compared to stage matched siblings. Based on the physiology of RB neurons in *maco* mutants, nicotine most likely initiated locomotion by activating spinal interneurons. However, in older mutants, nicotine-evoked locomotion was greatly reduced. This could occur if the number of nAChRs in the maturing spinal circuitry associated with activating locomotion has been downregulated, or possibly never upregulated in the first place.

An alteration in spinal circuit development may also manifest as an inability to swim upon nicotine exposure. For example, RB neurons make excitatory connections with interneurons in spinal cord associated with the generation of locomotion [22]. In *maco* mutants, RB neurons do not fire action potentials and thus cannot efficiently communicate with their interneuronal partners. If cellular communication of this type is required for the spinal interneurons to develop properly, they may not form synaptic connections correctly with their downstream partners. Over time, this could lead to the abnormal development of spinal circuits that simply cannot function well.

Normal RB activity may also influence the expression of proteins, including nAChRs in spinal interneurons. If interneurons do not receive the proper excitatory drive from RB neurons, they may not upregulate nAChR expression. Thus, the cellular and receptor substrates necessary for mediating nicotine-evoked locomotion in *maco* mutants may be altered as a result of having physiologically compromised RB neurons during the time when spinal circuits are developing. On the other hand, we cannot rule against the possibility that the DRG neurons, which express nAChRs, are also altered in the *maco* mutants.

Some characteristics of the onset of the NLR bear a noteworthy resemblance to the well-known touch-evoked response in zebrafish embryos, such as the swimming speed and tail beat frequency of these responses (Dr. Matthew E. Wolter, Svoboda laboratory, University of Wisconsin—Milwaukee; personal communication). Both responses can be elicited early enough in development (roughly 36 hpf) that presumably both responses utilize the same rudimentary anatomical structures of the developing embryo to elicit locomotor output. Moreover, the tail beat frequency of embryos in the NLR was very similar to the tail beat frequency of musculature bends in touch-evoked escapes (data not shown). It is important to note that in contrast to the younger embryos analyzed by Thomas and collaborators, 36 hpf and 48 hpf zebrafish only exhibited a modest motor output when exposed to 30μM nicotine. But when the concentration of nicotine was increased, the locomotor output was robust and happened very quickly. In these older fish, the ineffectiveness of 30μM nicotine to initiate locomotion is likely related to nicotine having to pass through the skin and penetrating more muscle and connective tissue than in the younger embryos. Increasing the concentration of nicotine ensures that nicotine will get to the neurons in spinal cord in a quick fashion.

The hyperlocomotor response observed in both free swimming 6 dpf zebrafish eleutheroembryos and 48 hpf embryos exposed to MeHg suggests that there is a common mechanism of MeHg-induced hyperactivity in both developmental stages. Furthermore, the effects observed at 6 dpf are likely the sequel of neurotoxic effects that occur at least as early in development as 48 hpf. Hyperactivity after embryonic MeHg insult has also been observed in rainbow trout (*Oncorhynchus mykiss*), largemouth bass (*Micropterus salmoides*) [23] and in rodents [24]. More recently, a link has been suggested between prenatal MeHg exposure and the onset of attention deficit hyperactivity disorder (ADHD) in humans [25]. It is worth noting that the lack of significant behavioral effects of MeHg in the highest dose utilized in our study (0.1μM) corresponds with the hormetic effects observed in the behavior of zebrafish embryos spawned by parents fed with MeHg-supplemented diets throughout their whole life span [13], in said study, embryos with the highest MeHg body burdens (0.6ppm) did not exhibit hyperactivity, while embryos with lower body burdens (≤0.2ppm) did.

The mechanisms by which MeHg causes the observed hyperactivity is unclear; in fact, even the more fundamental question of how exactly MeHg acts as a neurotoxicant remains unanswered [26]. However, our NLR assays in 48 hpf MeHg-exposed zebrafish embryos suggest that MeHg-induced hyperactivity is not associated with input from higher centers of the brain, but more likely to alterations in the spinal cord and the developing hindbrain. To the author’s knowledge, no previous studies have addressed the putative link between MeHg-mediated effects in the spinal cord and locomotor abnormalities. However, a link between oxidative stress in the cerebellum and hyperactivity has been observed in rodents [27]. It could be argued that locomotor effects in MeHg-exposed embryos during the NLR assay could be associated with effects of MeHg directly on nAChRs, as it has been reported that MeHg can bind to these receptors [28]; nevertheless, this is an unlikely scenario in our study, since the doses of MeHg utilized were not high enough to cause direct binding to nAChRs, furthermore, high throughput genomics in MeHg-exposed zebrafish embryos have not demonstrated effects of MeHg on the expression of the genes required to synthesize the proteins that comprise nAChRs (Mora-Zamorano, unpublished data).

Our approach also proved effective at quantifying brief episodes of locomotor bursts utilizing low-cost equipment. Other groups have developed excellent methodologies to quantify locomotor activity in multiple zebrafish [29]; however they rely on the usage of a high-speed camera, which can be a costly piece of equipment for a low budget laboratory. Regardless of the cellular and anatomical mechanisms of MeHg-induced behavioral alteration, the spontaneous-swimming and the NLR assay show promise as useful tools in behavioral toxicology screening.

## Supporting Information

S1 Fig Characteristic kinematics of the NLR in zebrafish embryos. The NLR is a characteristic locomotor response triggered by an exposure to a high concentration of nicotine (*e*.*g*. 30–240μM). This behavioral response is characterized by four phases: A) zebrafish embryos younger than 4 dpf do not exhibit free swimming, thus when exposed to a high nicotine concentration the embryos first remain immotile for a few seconds, while the nicotine penetrates the skin and muscle; B) once the nicotine is absorbed, the embryos abruptly initiate a vigorous and continuous locomotor burst that lasts several seconds, many times advancing in a spiraling trajectory; C) the locomotor response attenuates and many fish begin to erratically twitch without any forward propulsion; D) all embryos come to a complete halt.
(TIFF)

S2 Fig Custom-made behavior observation chamber. A) The behavior observation chamber consists of a manifold of Logitech c920 webcams that point downwards onto a tray with weigh boats that serve as arenas for the swimming embryos. The webcams are connected to a remote computer and the video footage is streamed using the MATLAB image acquisition toolbox. B) The ctrax tracking algorithm can quantify the locomotor activity of multiple fish embryos in the same arena simultaneously.
(TIFF)

S1 File Sample tracking video. This video is a representative example of an NLR assay trial after being analyzed with the ctrax machine vision algorithm.
(AVI)
