# Supplementary figures and images for: The Nicotine-Evoked Locomotor Response: A Behavioral Paradigm for Toxicity Screening in Zebrafish (Danio rerio) Embryos and Eleutheroembryos Exposed to Methylmercury
Source: PLoS One. 2016 Apr 28;11(4):e0154570. doi: 10.1371/journal.pone.0154570 (PMC4849578; doi:10.1371/journal.pone.0154570)

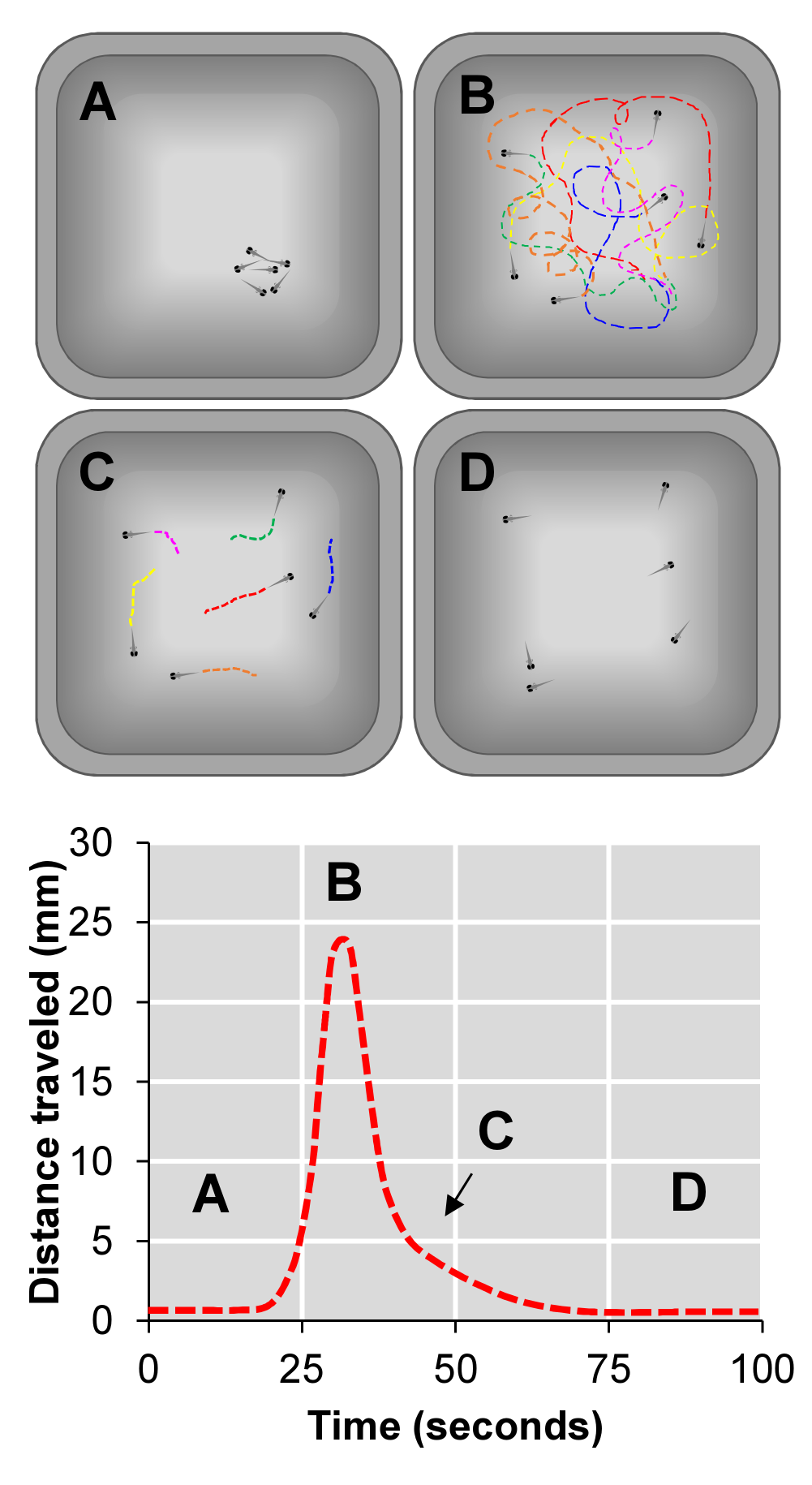

Supplement: S1 Fig — The NLR is a characteristic locomotor response triggered by an exposure to a high concentration of nicotine (e.g. 30–240μM). This behavioral response is characterized by four phases: A) zebrafish embryos younger than 4 dpf do not exhibit free swimming, thus when exposed to a high nicotine concentration the embryos first remain immotile for a few seconds, while the nicotine penetrates the skin and muscle; B) once the nicotine is absorbed, the embryos abruptly initiate a vigorous and continuous locomotor burst that lasts several seconds, many times advancing in a spiraling trajectory; C) the locomotor response attenuates and many fish begin to erratically twitch without any forward propulsion; D) all embryos come to a complete halt. (TIFF) [file pone.0154570.s001.tiff]

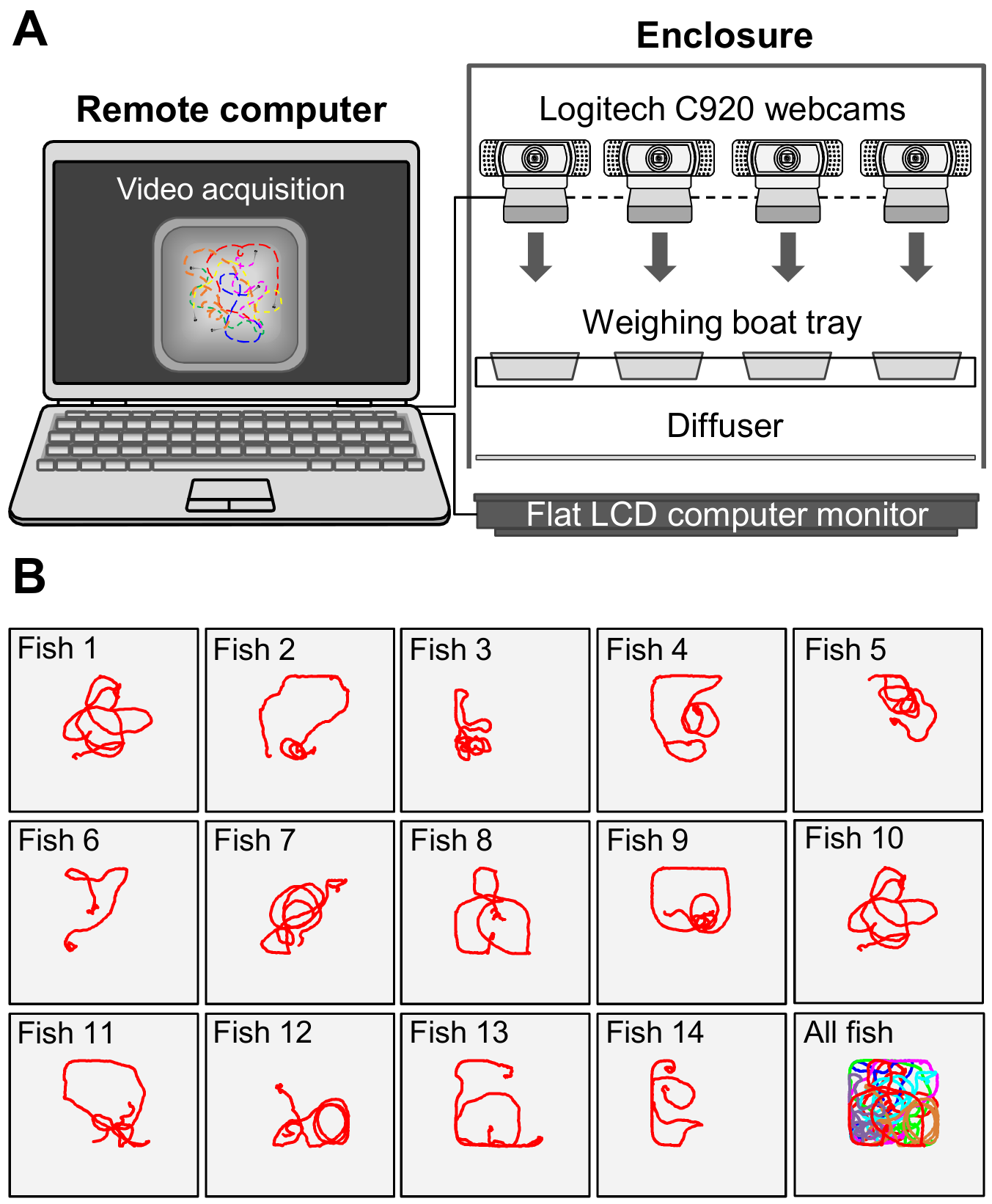

Supplement: S2 Fig — A) The behavior observation chamber consists of a manifold of Logitech c920 webcams that point downwards onto a tray with weigh boats that serve as arenas for the swimming embryos. The webcams are connected to a remote computer and the video footage is streamed using the MATLAB image acquisition toolbox. B) The ctrax tracking algorithm can quantify the locomotor activity of multiple fish embryos in the same arena simultaneously. (TIFF) [file pone.0154570.s002.tiff]
